# Supplementary material for: Lipopeptide Epimers and a Phthalide Glycerol Ether with AChE Inhibitory Activities from the Marine-Derived Fungus Cochliobolus Lunatus SCSIO41401
Source: Mar Drugs. 2020 Oct 30;18(11):547. doi: 10.3390/md18110547 (PMC7693918; doi:10.3390/md18110547)
Supplement: Supplementary file 1 [file marinedrugs-18-00547-s001.pdf]

Supporting Information

Lipopeptide Epimers and a Phthalide Glycerol Ether with AChE Inhibitory Activities from the Marine-Derived Fungus *Cochliobolus Lunatus* SCSIO41401

Yu Dai [1,†], Kunlong Li [1,2,†], Jianglian She [1], Yanbo Zeng [3], Hao Wang [3], Shengrong Liao [1,2], Xiuping Lin [1,2], Bin Yang [1,2], Junfeng Wang [1,2], Huaming Tao [4], Haofu Dai [3], Xuefeng Zhou [1,2,*] and Yonghong Liu [1,2,5,*]

[1] CAS Key Laboratory of Tropical Marine Bio-resources and Ecology, Guangdong Key Laboratory of Marine Materia Medica, South China Sea Institute of Oceanology, Chinese Academy of Sciences, Guangzhou, 510301, China; daiyu15@mails.ucas.ac.cn (Y.D.); likunlong16@mails.ucas.edu.cn (K.L.); shejianglian20@mails.ucas.ac.cn (J.S.); ljrss@126.com (S.L.); xiupinglin@hotmail.com (X.L.); yangbin@scsio.ac.cn (B.Y.); wangjunfeng@scsio.ac.cn (J.W.)

[2] Southern Marine Science and Engineering Guangdong Laboratory (Guangzhou), Guangzhou, 511458, China

[3] Research and Development of Natural Product from Li Folk Medicine, Institute of Tropical Bioscience and Biotechnology, Chinese Academy of Tropical Agriculture Sciences, Haikou 571101, China; zengyanbo@itbb.org.cn (Y.Z.); wanghao@itbb.org.cn (H.W.); daihaofu@itbb.org.cn (H.D.)

[4] School of Traditional Chinese Medicine, Southern Medical University, Guangzhou 510515, China; taohm@smu.edu.cn

[5] Wuya College of Innovation, Shenyang Pharmaceutical University, Shenyang 110016, China

* Correspondence: xfzhou@scsio.ac.cn (X.Z.); yonghongliu@scsio.ac.cn (Y.L.)

† These authors contributed equally to this work.

List of supporting information

Figure S1. The ^{1}H-NMR spectrum of **1** in CD_3OD 4
Figure S2. The ^{13}C-NMR spectrum of **1** in CD_3OD 4
Figure S3. The DEPT 135 NMR spectrum of **1** in CD_3OD 5
Figure S4. The HSQC spectrum of **1** in CD_3OD 5
Figure S5. The HMBC spectrum of **1** in CD_3OD 6
Figure S6. The COSY spectrum of **1** in CD_3OD 6
Figure S7. The ROESY spectrum of **1** in CD_3OD 7
Figure S8. The UV spectrum of **1** 7
Figure S9. The (+)-HRESIMS spectrum of **1** 8
Figure S10. The ^{1}H-NMR spectrum of **2** in CD_3OD 8
Figure S11. The ^{13}C-NMR spectrum of **2** in CD_3OD 9
Figure S12. The DEPT 135 NMR spectrum of **2** in CD_3OD 9
Figure S13. The HSQC spectrum of **2** in CD_3OD 10
Figure S14. The HMBC spectrum of **2** in CD_3OD 10
Figure S15. The COSY spectrum of **2** in CD_3OD 11
Figure S16. The ROESY spectrum of **2** in CD_3OD 11
Figure S17. The UV spectrum of **2** 12
Figure S18. The (+)-HRESIMS spectrum of **2** 13
Figure S19. The ^{1}H-NMR spectrum of **3** in CD_3OD 13
Figure S20. The ^{13}C-NMR spectrum of **3** in CD_3OD 13
Figure S21. The DEPT 135 NMR spectrum of **3** in CD_3OD 14
Figure S22. The HSQC spectrum of **3** in CD_3OD 14
Figure S23. The HMBC spectrum of **3** in CD_3OD 15
Figure S24. The ^{1}H-^{1}H COSY spectrum of **3** in CD_3OD 15
Figure S25. The UV spectrum of **3** 16
Figure S26. The (+)-HRESIMS spectrum of **3** 16
Figure S27. The ^{1}H-NMR spectrum of **4** in CD_3OD 17
Figure S28. The ^{13}C-NMR spectrum of **4** in CD_3OD 17
Figure S29. The ^{1}H-NMR spectrum of **5** in CD_3OD 18
Figure S30. The ^{13}C-NMR spectrum of **5** in CD_3OD 18

Figure S31. The ^{1}H-NMR spectrum of **6** in CD_3OD 19
Figure S32. The ^{13}C-NMR spectrum of **6** in CD_3OD 19
Figure S33. Preliminary Lineweaver–Burk plots for the AChE inhibition with **1**..…20

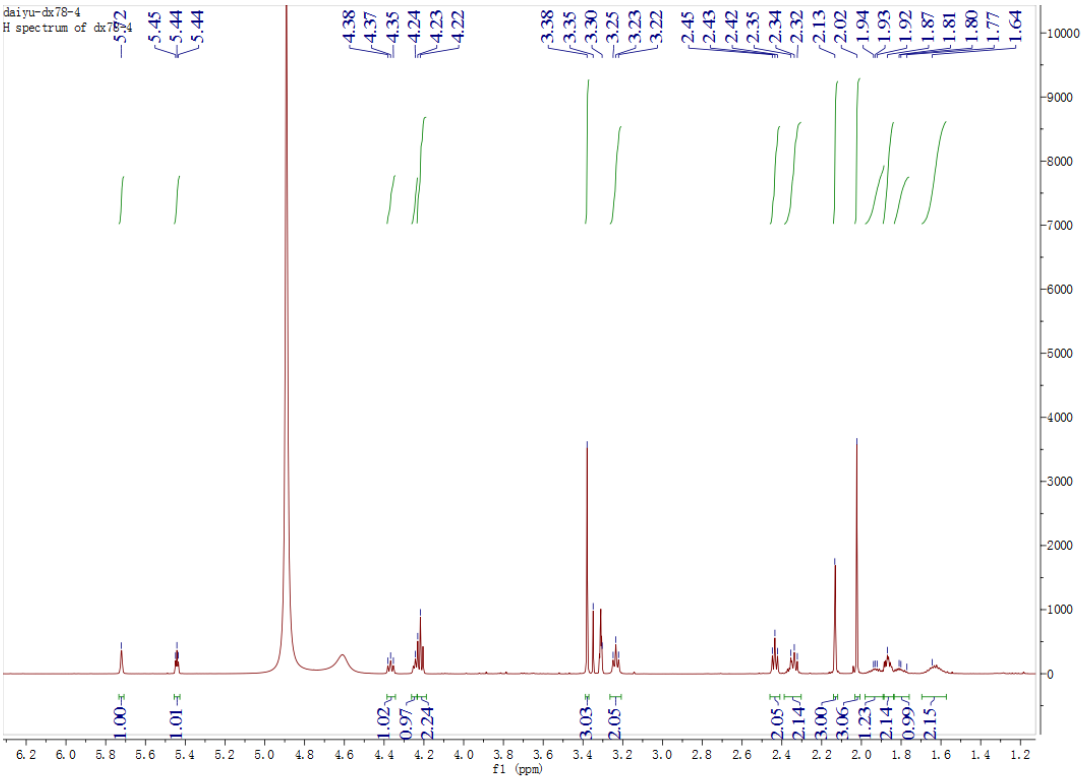

Figure S1. The ^{1}H-NMR spectrum of **1** in CD_3OD

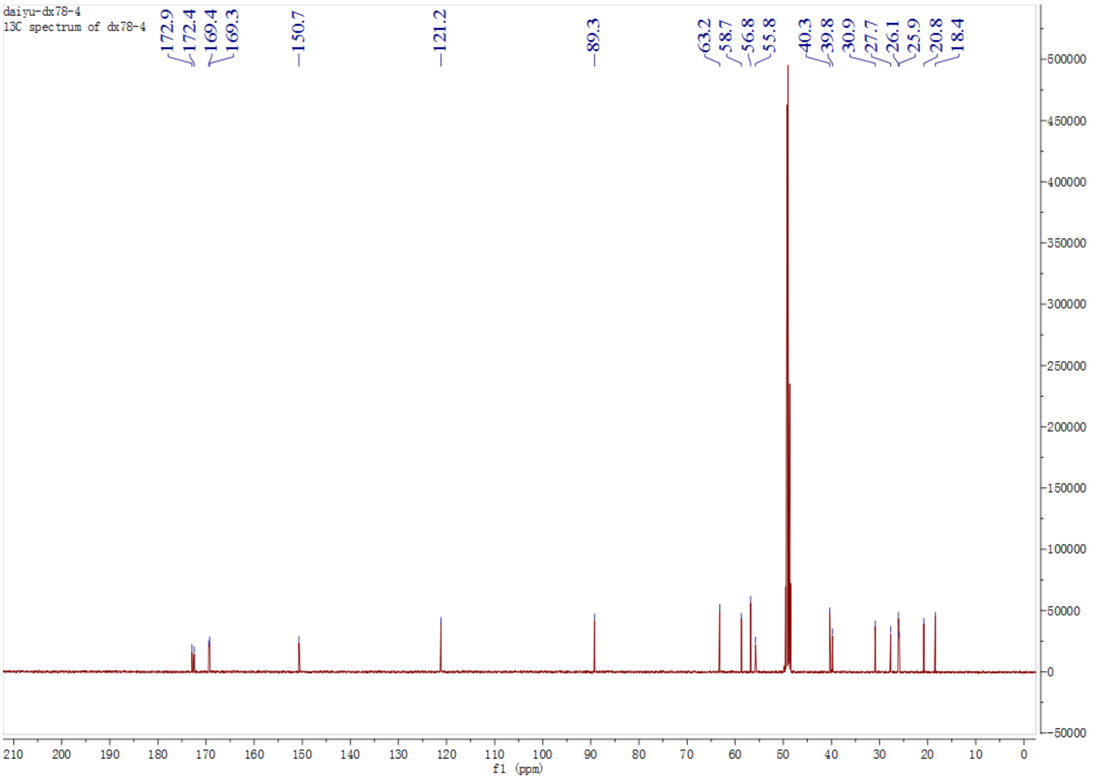

Figure S2. The ^{13}C-NMR spectrum of **1** in CD_3OD

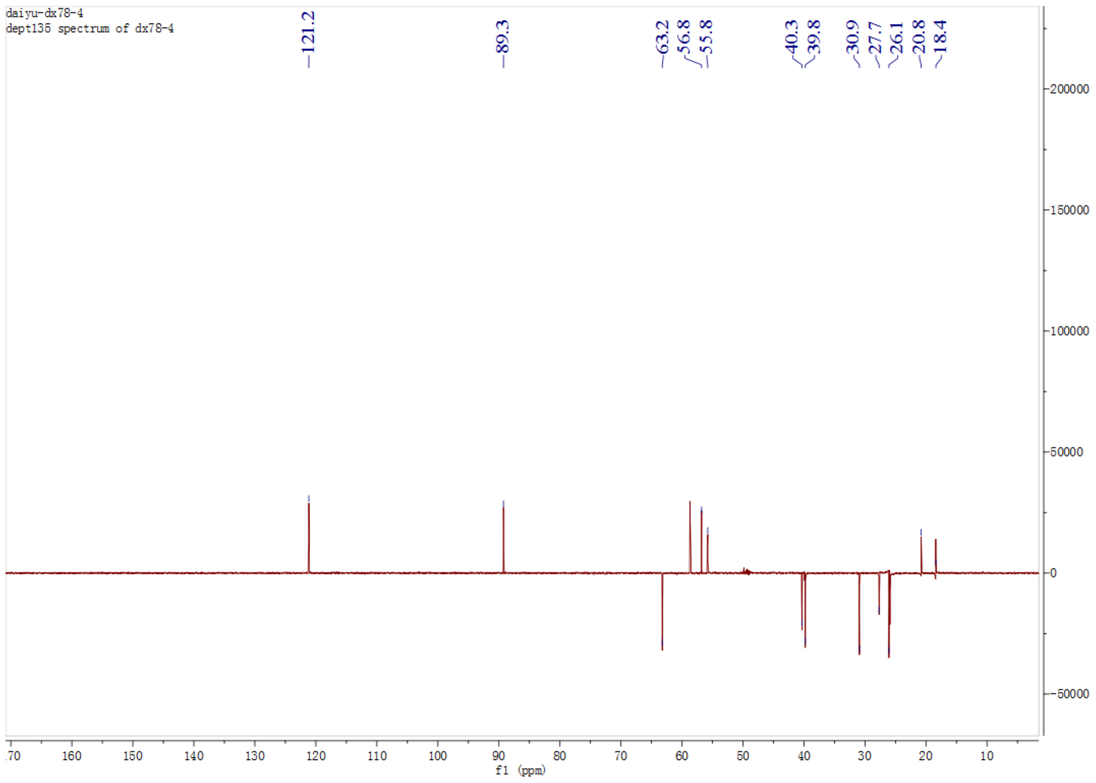

Figure S3. The DEPT 135 NMR spectrum of **1** in CD_3OD

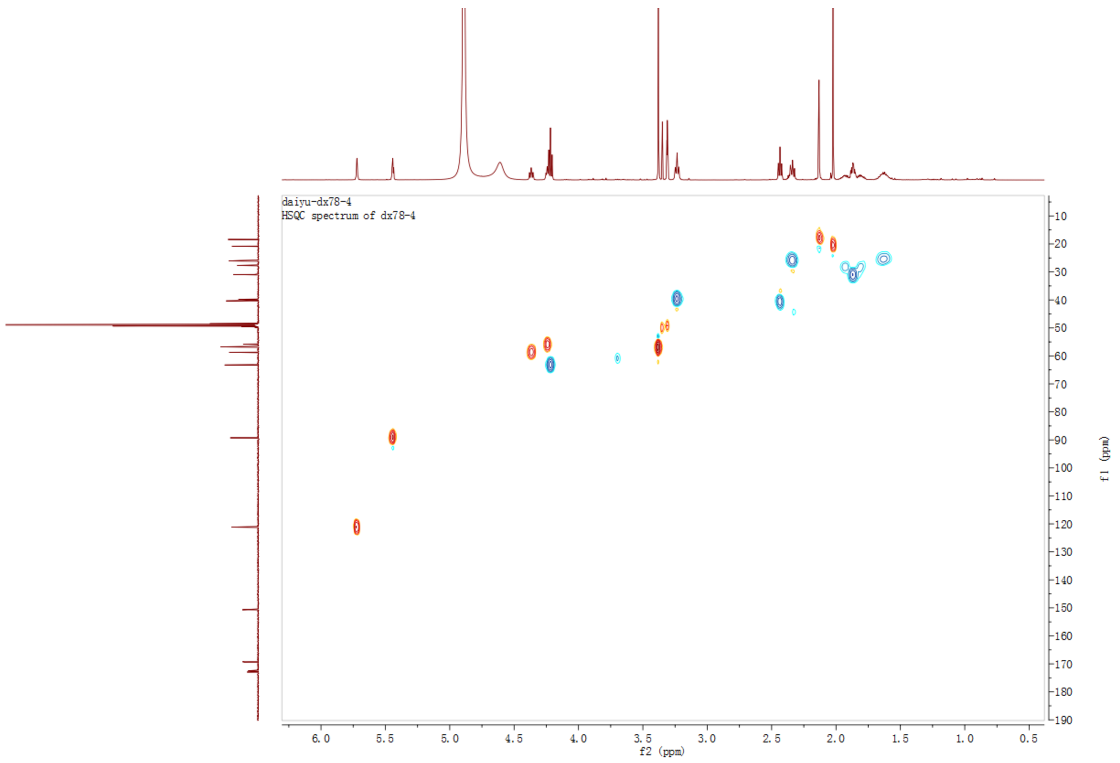

Figure S4. The HSQC spectrum of **1** in CD_3OD

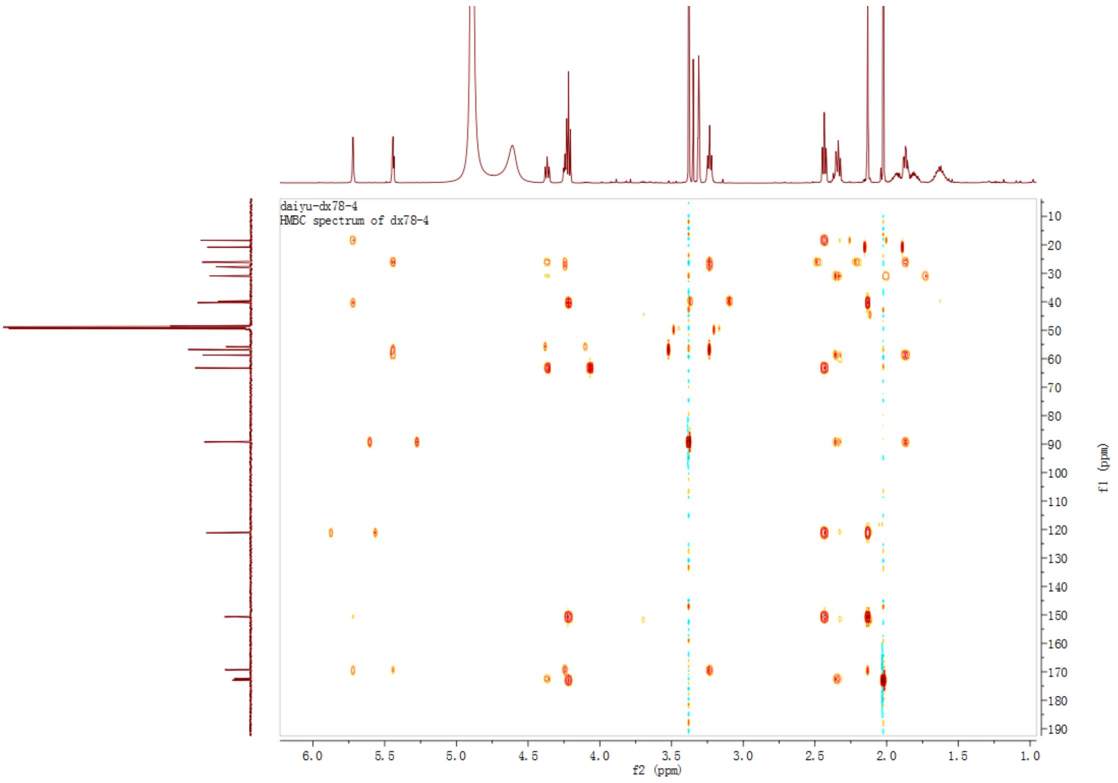

Figure S5. The HMBC spectrum of **1** in CD_3OD

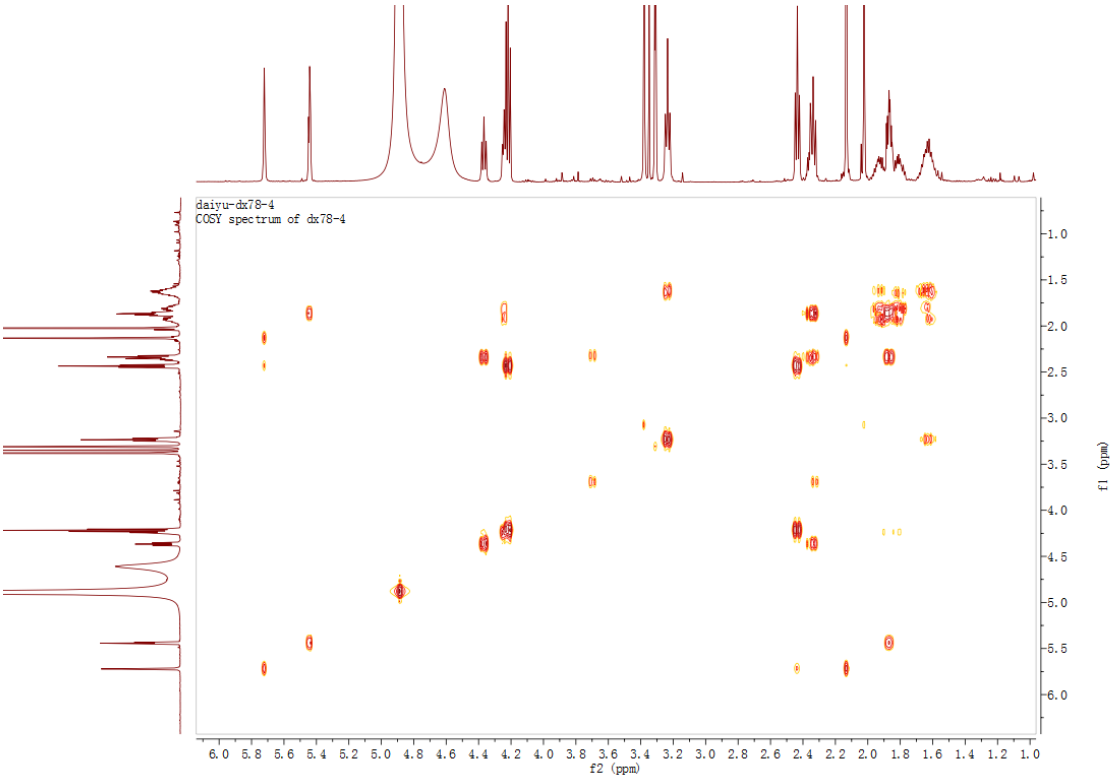

Figure S6. The COSY spectrum of **1** in CD_3OD

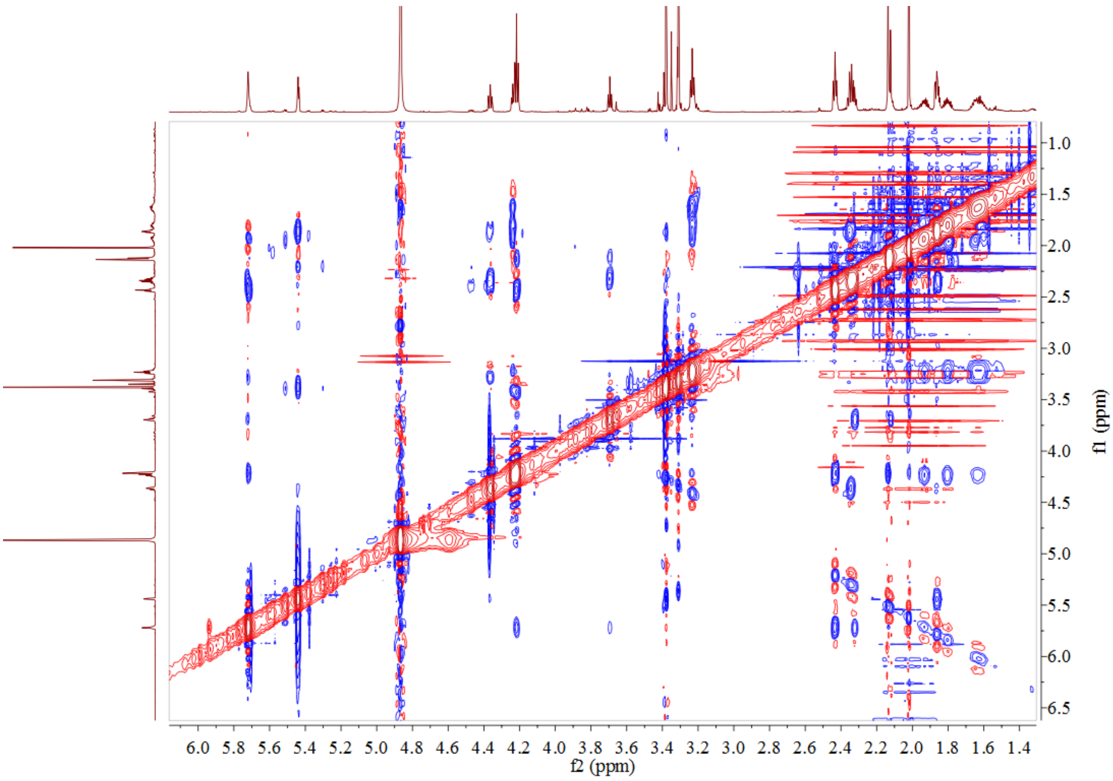

Figure S7. The ROESY spectrum of **1** in CD_3OD

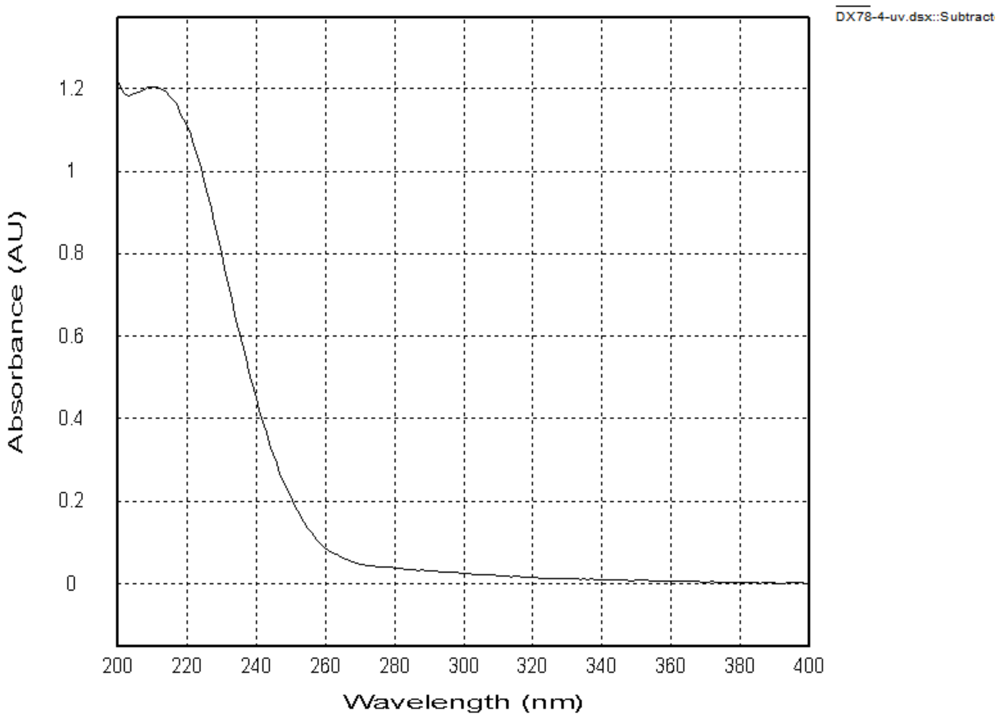

Figure S8. The UV spectrum of **1**

Mass Spectrum SmartFormula Report

Analysis Info

Analysis Name	D:\Data\MS\data\201804\guocui_dx78-4_pos_21_01_4507.d	Acquisition Date	4/3/2018 12:01:57 PM
Method	LC_Direct Infusion_pos_70-500mz.m	Operator	SCSIO
Sample Name	guocui_dx78-4_pos	Instrument	maXis 255552.00029
Comment			

Acquisition Parameter

Source Type	ESI	Ion Polarity	Positive	Set Nebulizer	0.4 Bar
Focus	Active	Set Capillary	4500 V	Set Dry Heater	180 °C
Scan Begin	70 m/z	Set End Plate Offset	-500 V	Set Dry Gas	4.0 l/min
Scan End	1500 m/z	Set Charging Voltage	0 V	Set Divert Valve	Waste
		Set Corona	0 nA	Set APCI Heater	0 °C

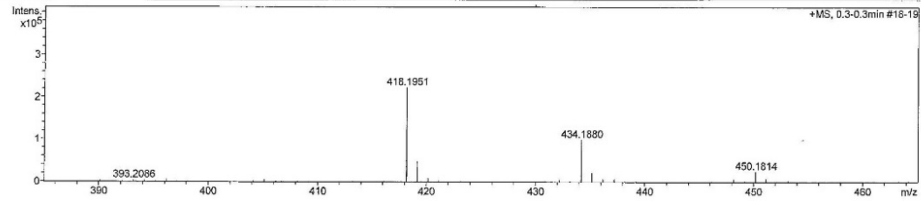

Meas. m/z	#	Ion Formula	Score	m/z	err [ppm]	err [mDa]	mSigma	rdb	e⁻ Conf	N-Rule
418.1951	1	C19H29N3NaO6	100.00	418.1949	-0.6	-0.2	4.8	6.5	even	ok
434.1880	1	C19H29N3NaO7	65.54	434.1898	-4.2	-1.8	2.9	6.5	even	ok
450.1814	1	C19H29N3NaO8	15.02	450.1847	-7.3	-3.3	9.3	6.5	even	ok
813.4000	1	C38H58N6NaO12	100.00	813.4005	0.6	0.5	12.6	12.5	even	ok
845.3884	1	C38H58N6NaO14	50.63	845.3903	-2.3	-1.9	16.3	12.5	even	ok
877.3763	1	C38H58N6NaO16	12.03	877.3802	4.4	3.8	25.1	12.5	even	ok

guocui_dx78-4_pos_21_01_4507.d

Bruker Compass DataAnalysis 4.1 printed: 4/4/2018 10:00:01 AM by: SCSIO Page 1 of 1

Figure S9. The (+)-HRESIMS spectrum of **1**

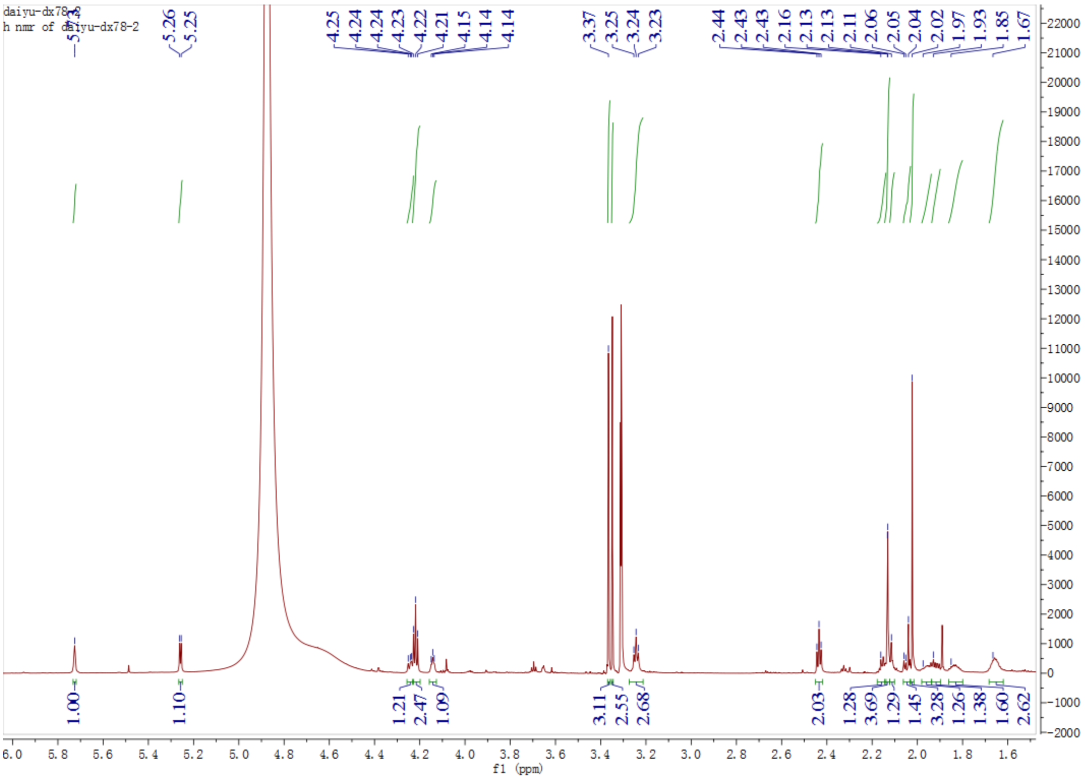

Figure S10. The ^{1}H-NMR spectrum of **2** in CD_3OD

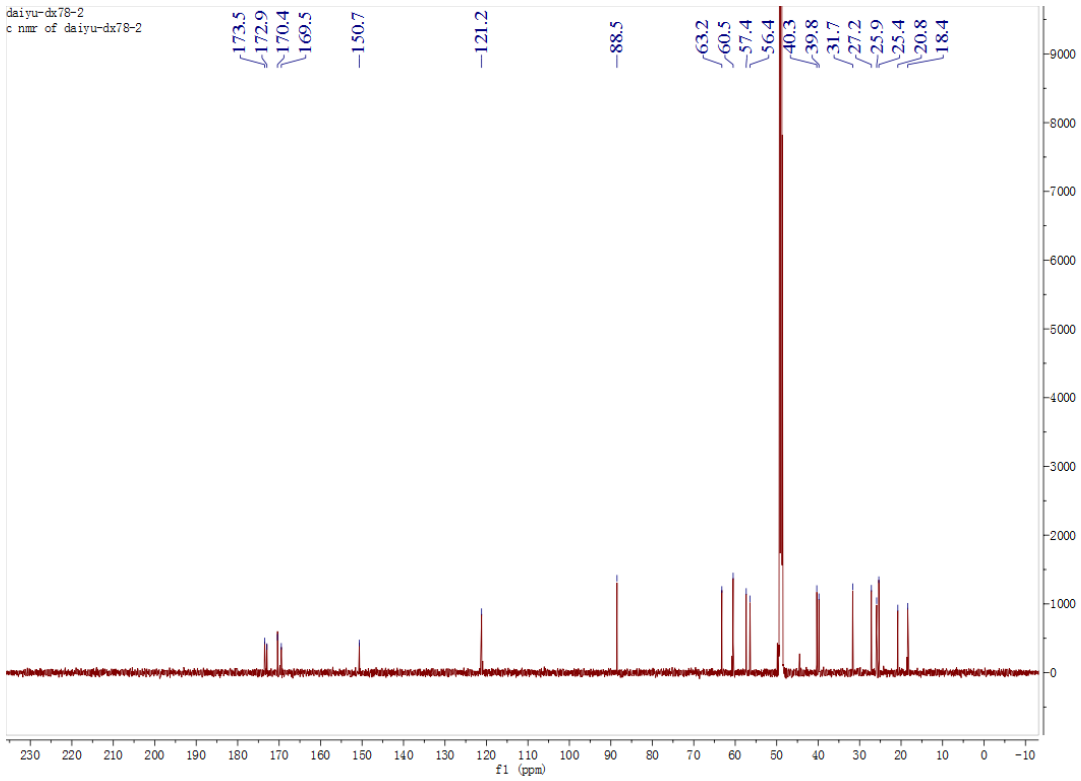

Figure S11. The ^{13}C-NMR spectrum of **2** in CD_3OD

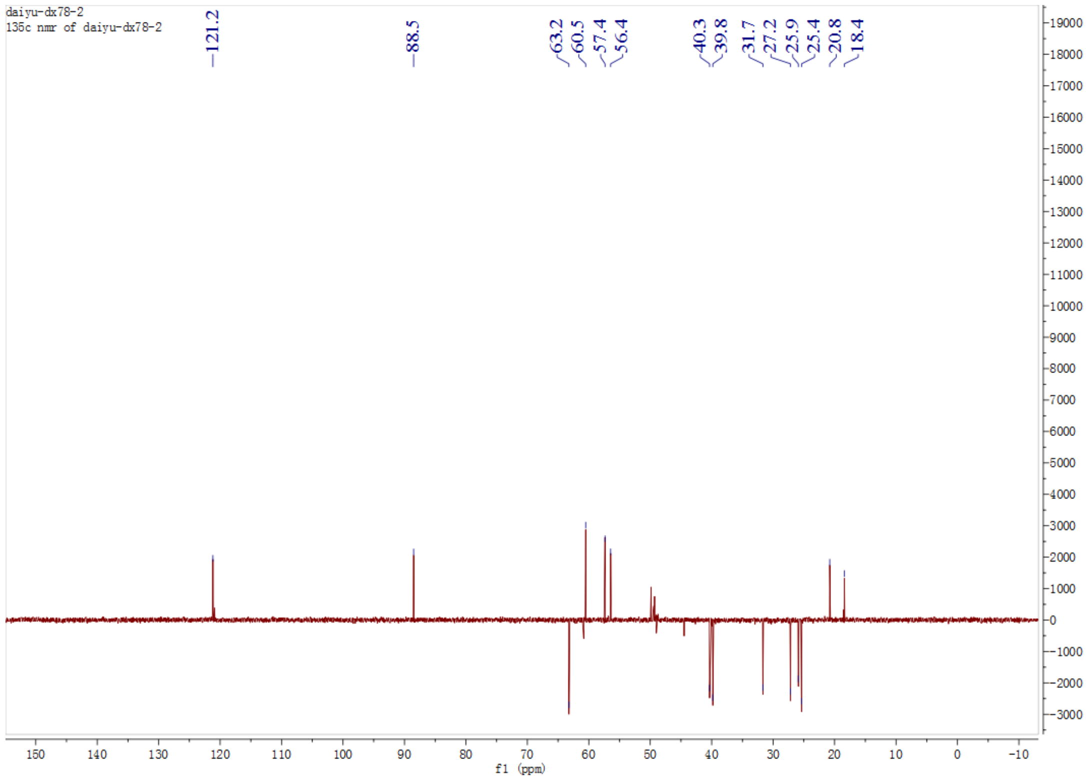

Figure S12. The DEPT 135 NMR spectrum of **2** in CD_3OD

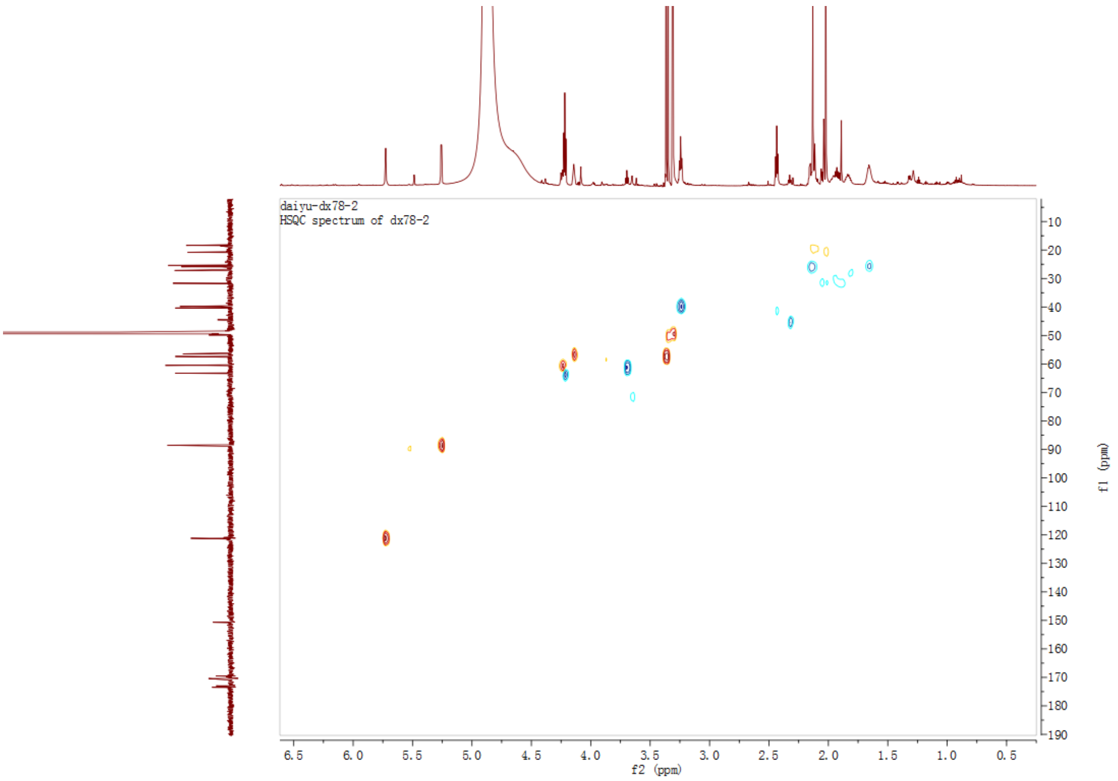

Figure S13. The HSQC spectrum of **2** in CD_3OD

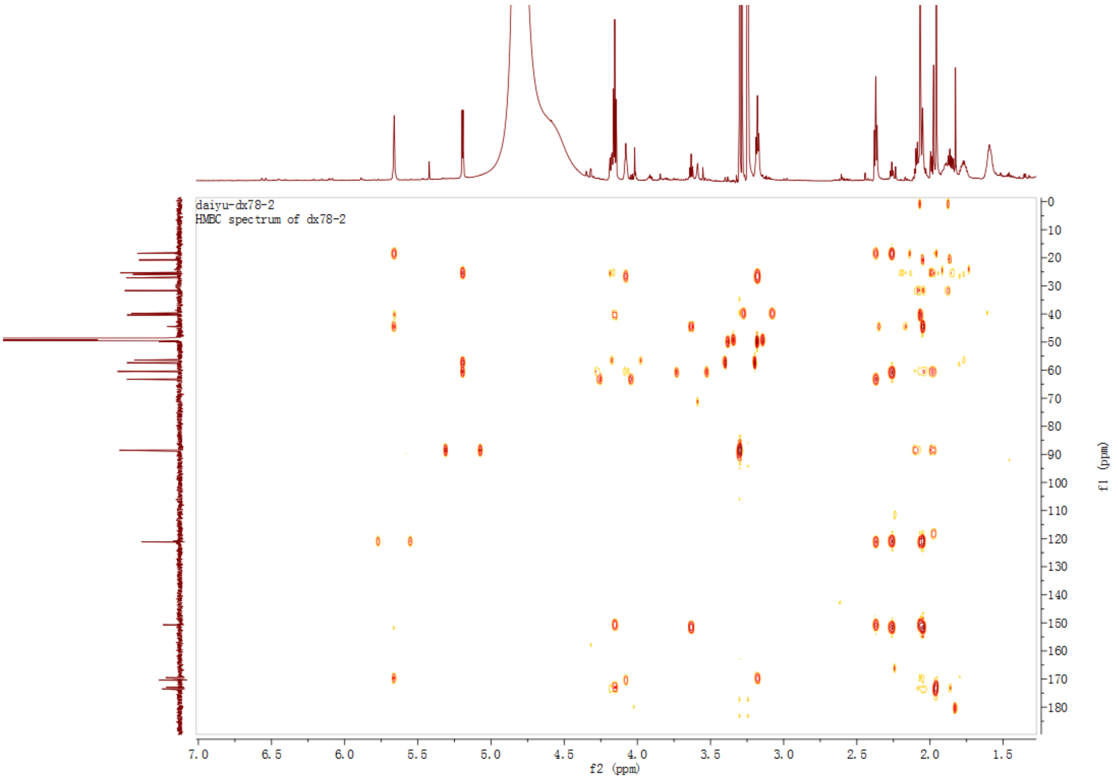

Figure S14. The HMBC spectrum of **2** in CD_3OD

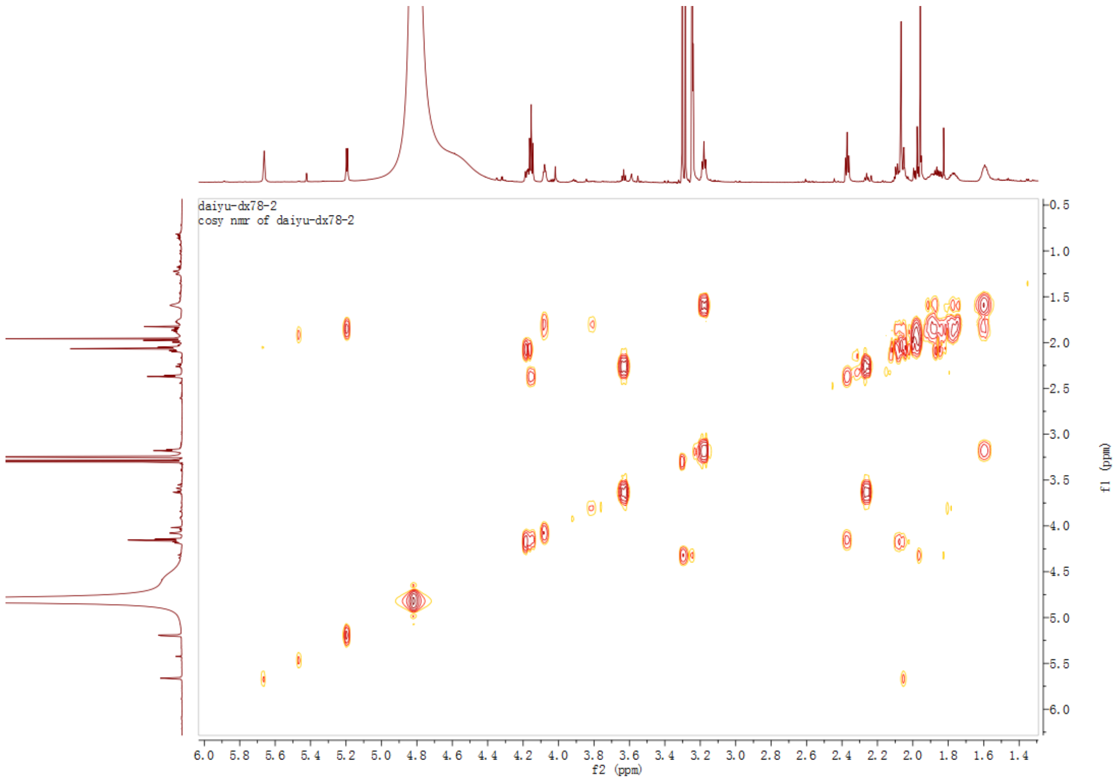

Figure S15. The COSY spectrum of **2** in CD_3OD

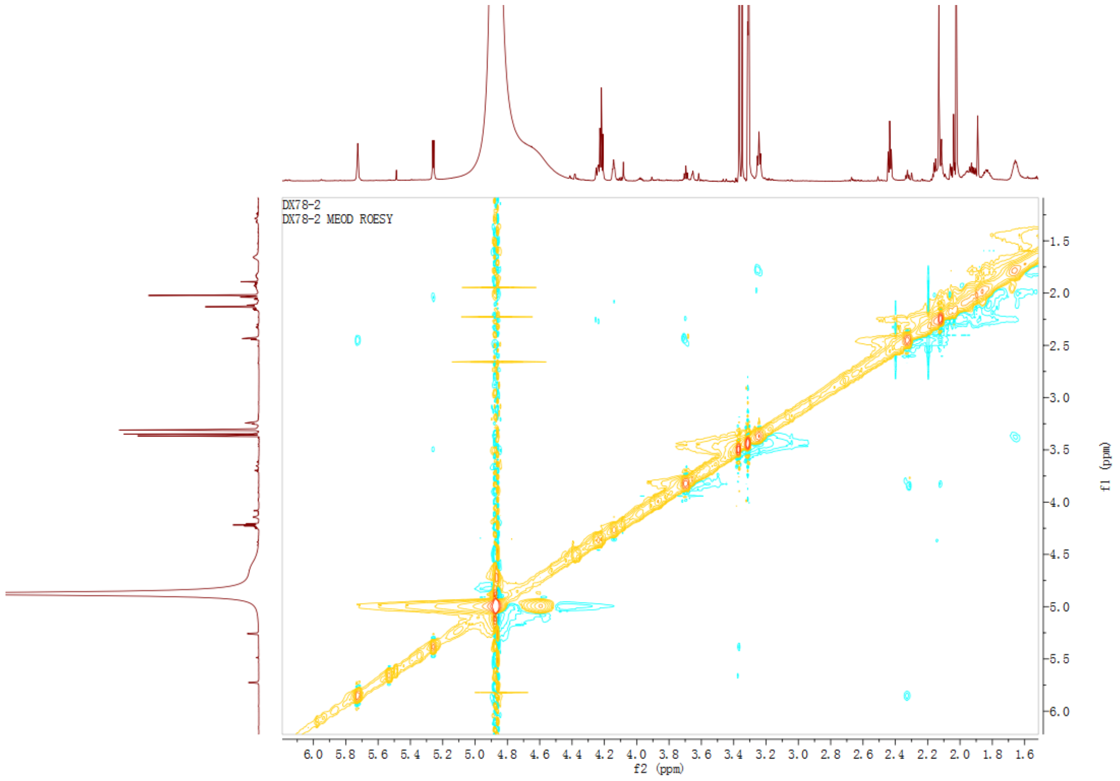

Figure S16. The ROESY spectrum of **2** in CD_3OD

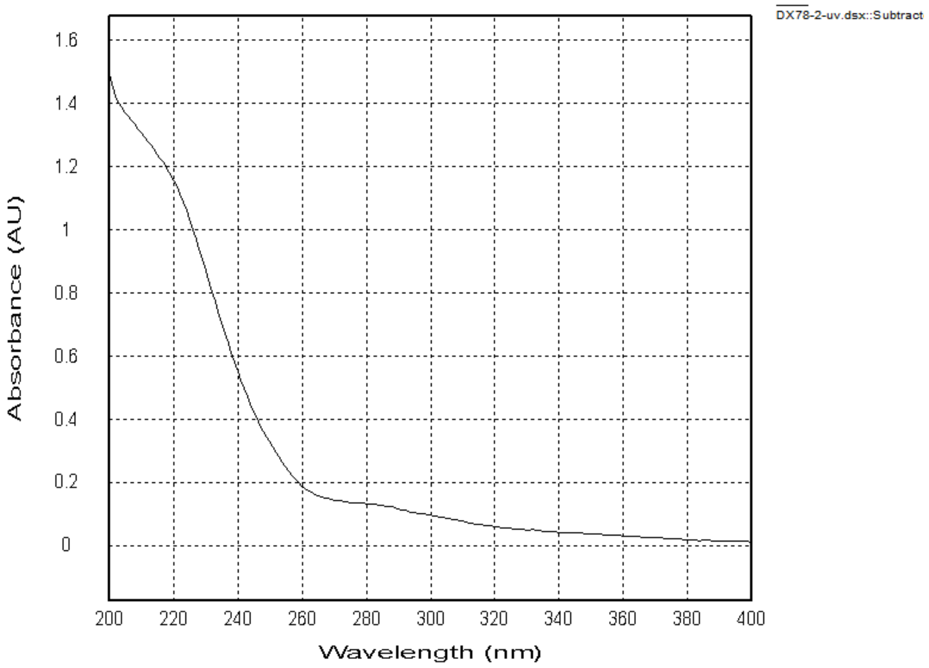

Figure S17. The UV spectrum of **2**

Mass Spectrum SmartFormula Report

Analysis Info

Analysis Name	D:\Data\MS\data\201804\daiyu_dx78-2_pos_31_01_4622.d	Acquisition Date	4/23/2018 2:33:33 PM	
Method	LC_Direct Infusion_pos_70-500mz.m	Operator	SCSIO	
Sample Name	daiyu_dx78-2_pos	Instrument	maXis	255552.00029
Comment				

Acquisition Parameter

Source Type	ESI	Ion Polarity	Positive	Set Nebulizer	0.4 Bar
Focus	Active	Set Capillary	4500 V	Set Dry Heater	180 °C
Scan Begin	70 m/z	Set End Plate Offset	-500 V	Set Dry Gas	4.0 l/min
Scan End	1500 m/z	Set Charging Voltage	0 V	Set Divert Valve	Waste
		Set Corona	0 nA	Set APCI Heater	0 °C

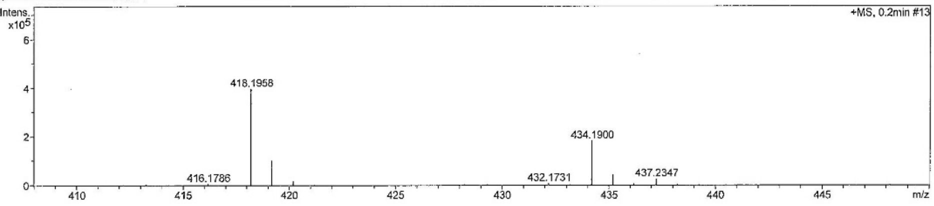

Meas. m/z	#	Ion Formula	Score	m/z	err [ppm]	err [mDa]	mSigma	rdb	e⁻ Conf	N-Rule
418.1958	1	C19H29N3NaO6	100.00	418.1949	2.2	0.9	21.7	6.5	even	ok
434.1900	1	C19H29N3NaO7	100.00	434.1898	-0.4	-0.2	10.5	6.5	even	ok
829.3949	1	C38H58N6NaO13	100.00	829.3954	0.6	0.5	18.3	12.5	even	ok
845.3891	1	C38H58N6NaO14	100.00	845.3903	1.5	1.3	15.1	12.5	even	ok

daiyu_dx78-2_pos_31_01_4622.d

Bruker Compass DataAnalysis 4.1 printed: 4/23/2018 2:45:11 PM by: SCSIO Page 1 of 1

Figure S18. The (+)-HRESIMS spectrum of **2**

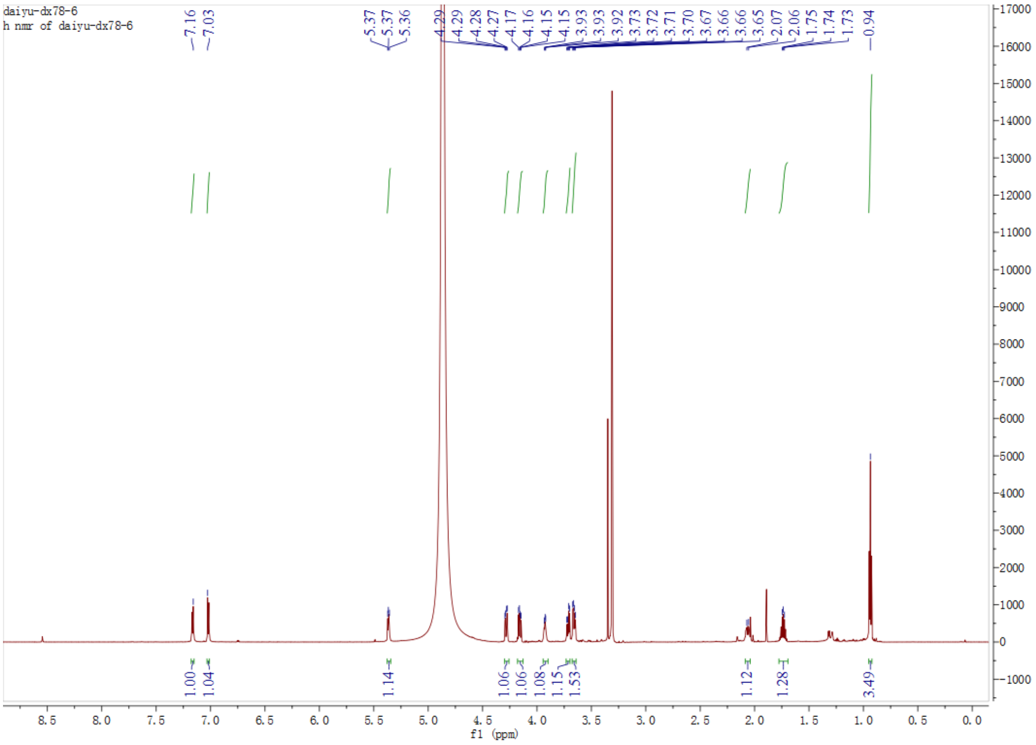

Figure S19. The ^{1}H-NMR spectrum of **3** in CD_3OD

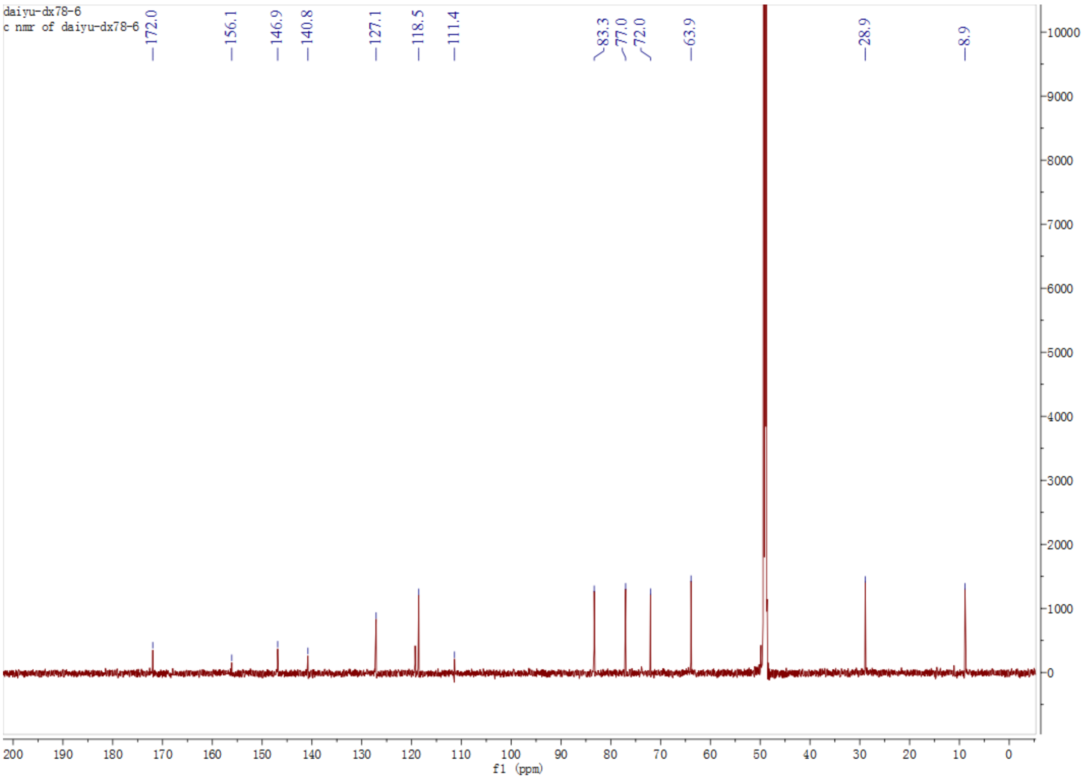

Figure S20. The ^{13}C-NMR spectrum of **3** in CD_3OD

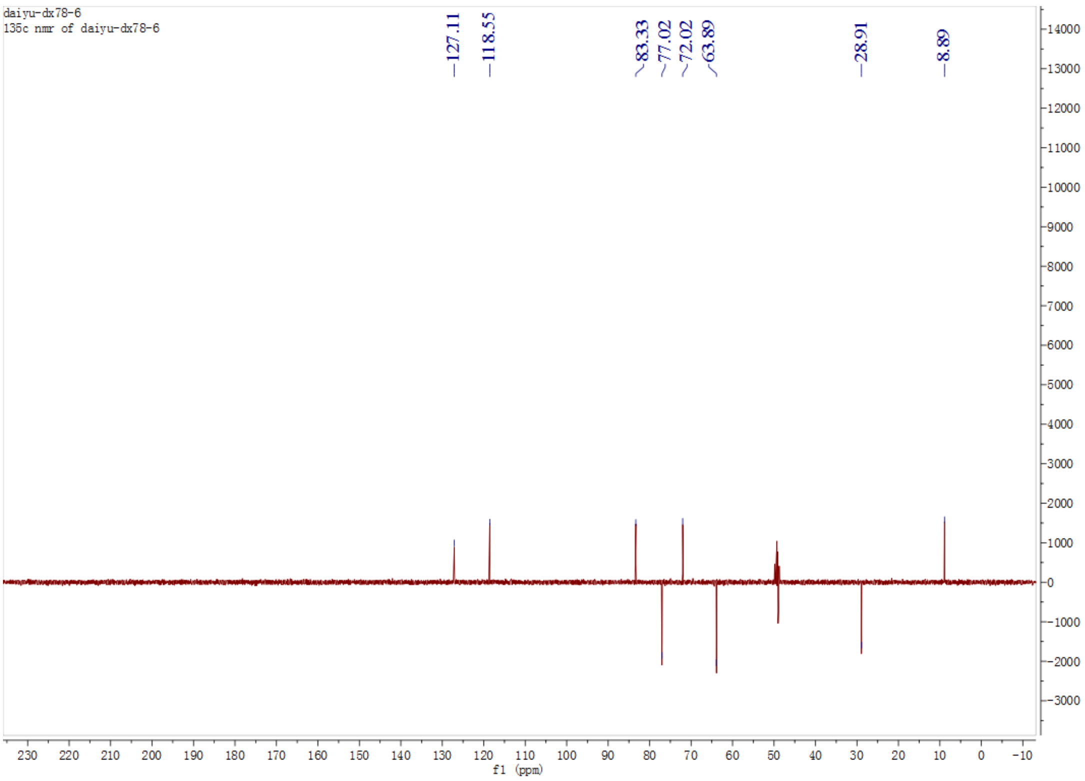

Figure S21. The DEPT 135 NMR spectrum of **3** in CD_3OD

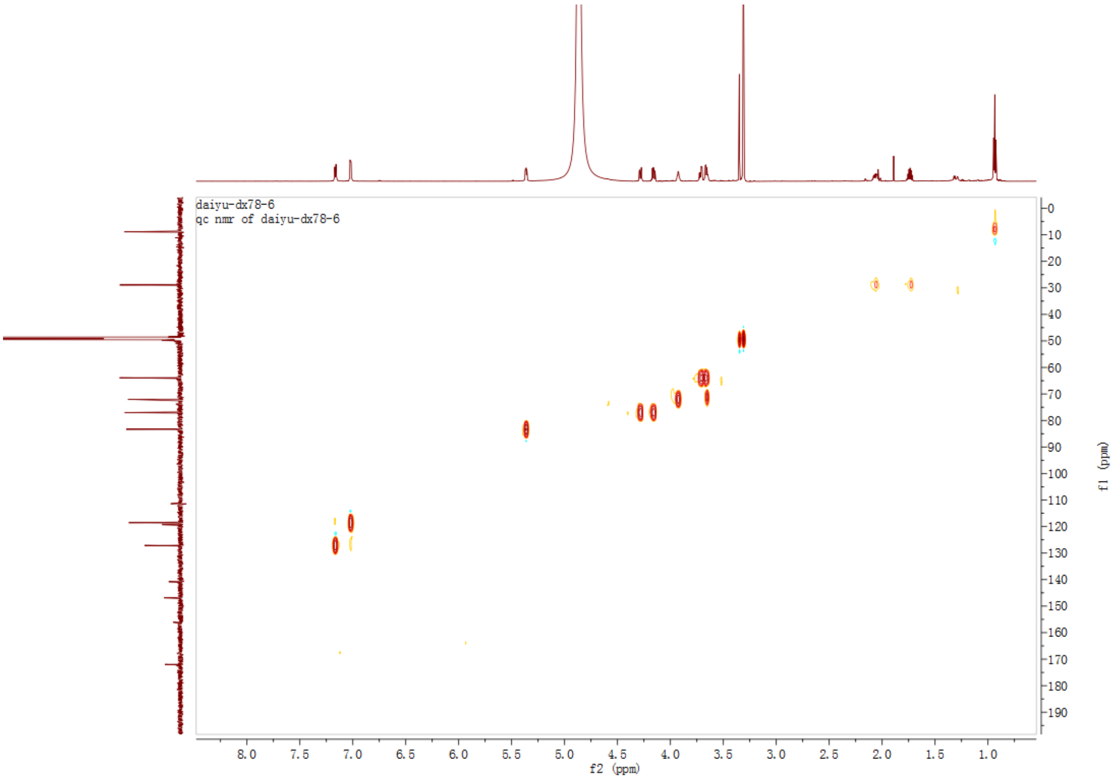

Figure S22. The HSQC spectrum of **3** in CD_3OD

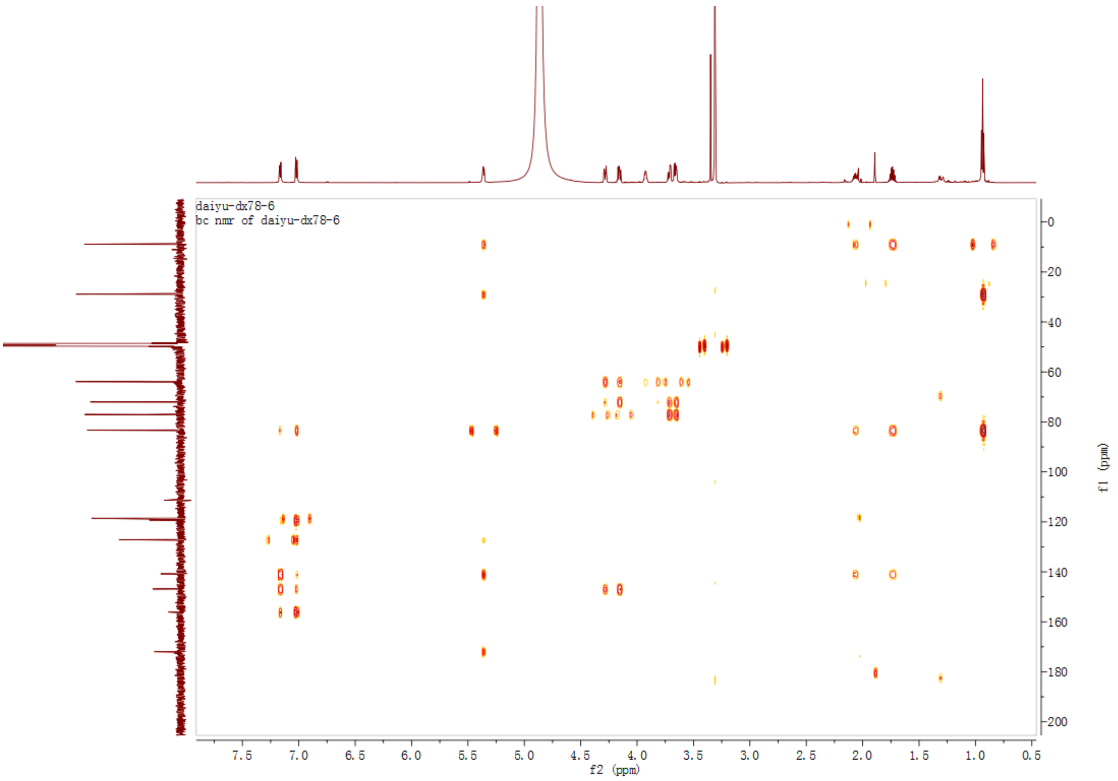

Figure S23. The HMBC spectrum of **3** in CD3OD

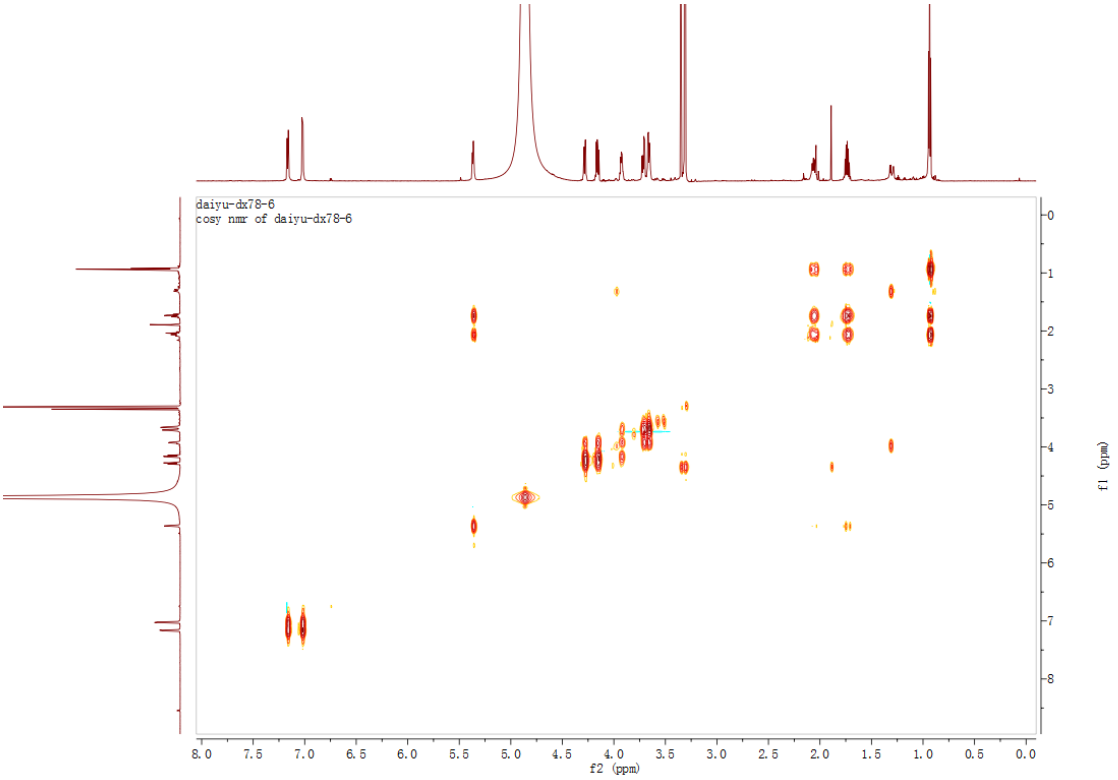

Figure S24. The ^{1}H-^{1}H COSY spectrum of **3** in CD3OD

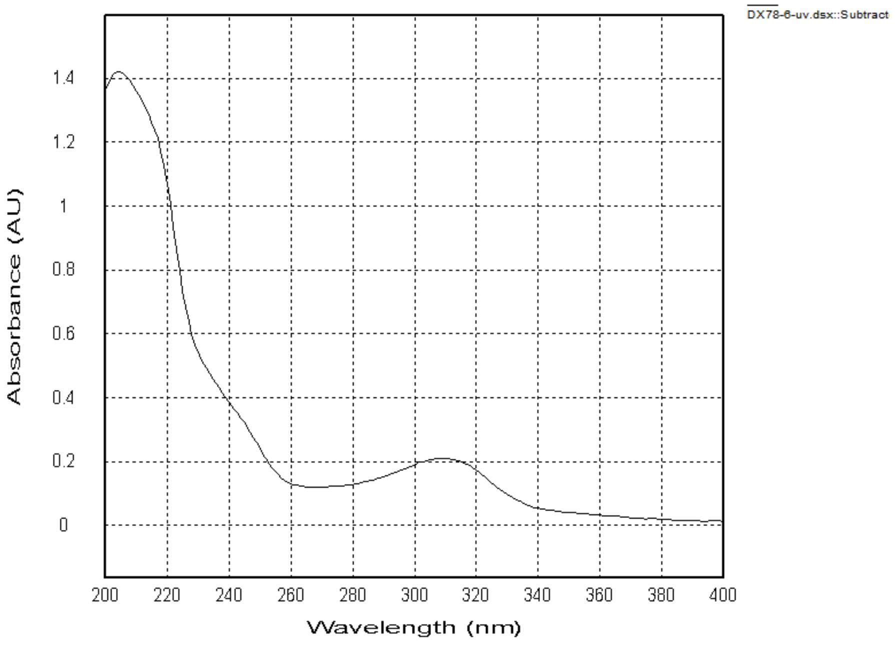

Figure S25. The UV spectrum of **3**

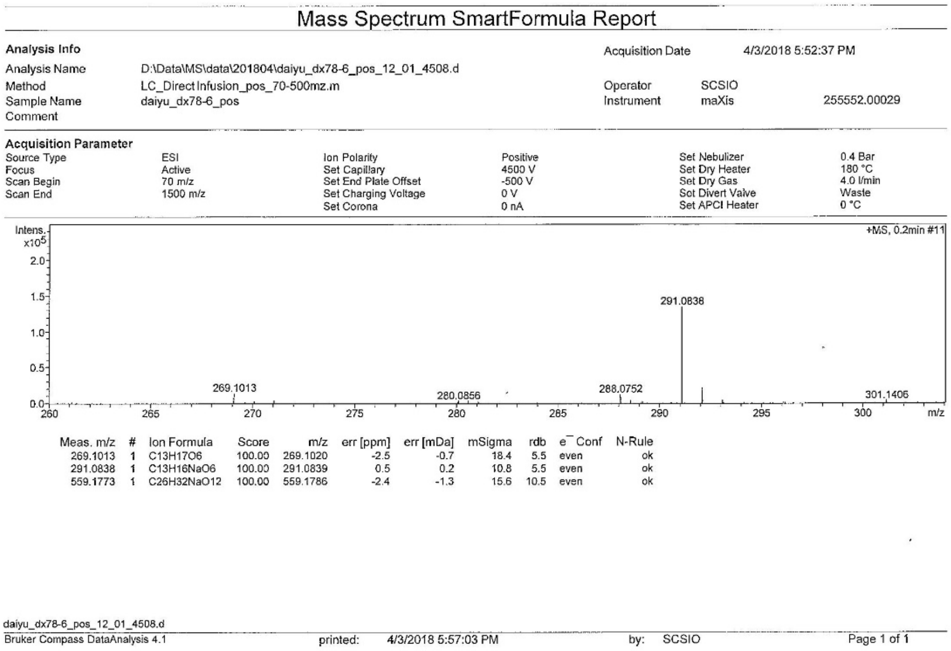

Mass Spectrum SmartFormula Report

Analysis Info

Analysis Name	D:\Data\MS\data\201804\daiyu_dx78-6_pos_12_01_4508.d	Acquisition Date	4/3/2018 5:52:37 PM
Method	LC_Direct Infusion_pos_70-500mz.m	Operator	SCSIO
Sample Name	daiyu_dx78-6_pos	Instrument	maXis 255552.00029
Comment			

Acquisition Parameter

Source Type	ESI	Ion Polarity	Positive	Set Nebulizer	0.4 Bar
Focus	Active	Set Capillary	4500 V	Set Dry Heater	180 °C
Scan Begin	70 m/z	Set End Plate Offset	-500 V	Set Dry Gas	4.0 l/min
Scan End	1500 m/z	Set Charging Voltage	0 V	Sot Divert Valve	Waste
		Set Corona	0 nA	Set APCI Heater	0 °C

Meas. m/z	#	Ion Formula	Score	m/z	err [ppm]	err [mDa]	mSigma	rdb	e⁻ Conf	N-Rule
269.1013	1	C13H17O6	100.00	269.1020	-2.5	-0.7	18.4	5.5	even	ok
291.0838	1	C13H16NaO6	100.00	291.0839	0.5	0.2	10.8	5.5	even	ok
559.1773	1	C26H32NaO12	100.00	559.1786	-2.4	-1.3	15.6	10.5	even	ok

daiyu_dx78-6_pos_12_01_4508.d
Bruker Compass DataAnalysis 4.1 printed: 4/3/2018 5:57:03 PM by: SCSIO Page 1 of 1

Figure S26. The (+)-HRESIMS spectrum of **3**

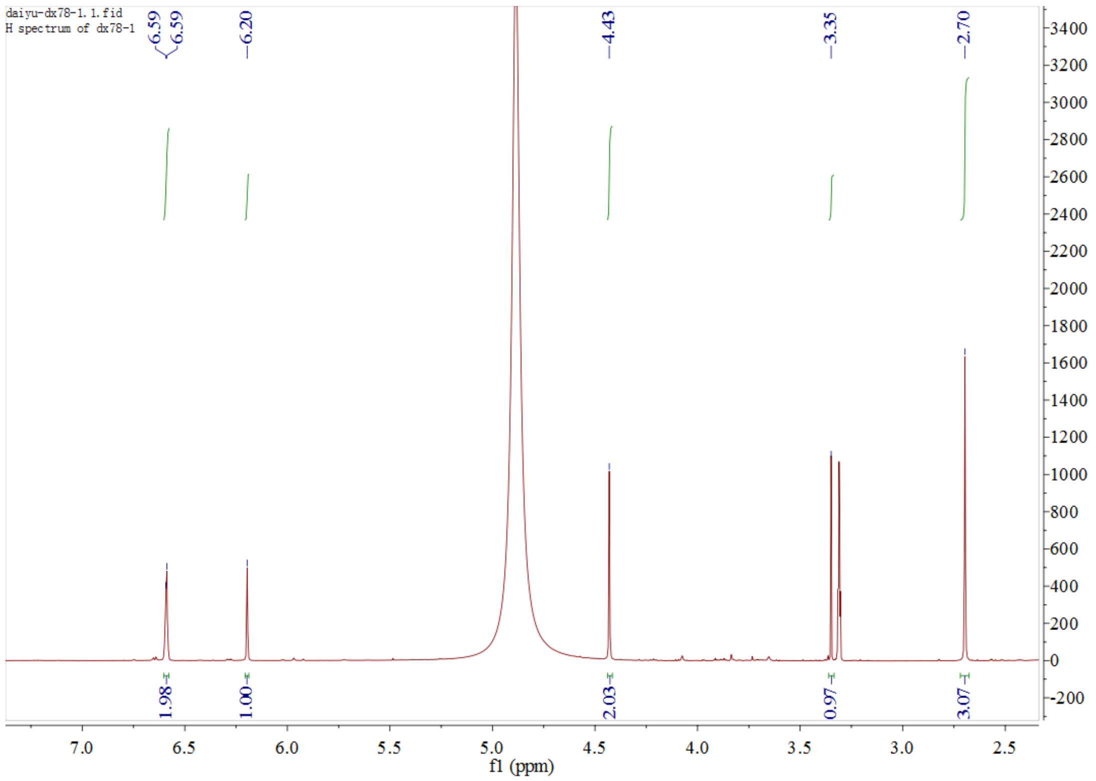

Figure S27. The ^{1}H-NMR spectrum of **4** in CD_3OD

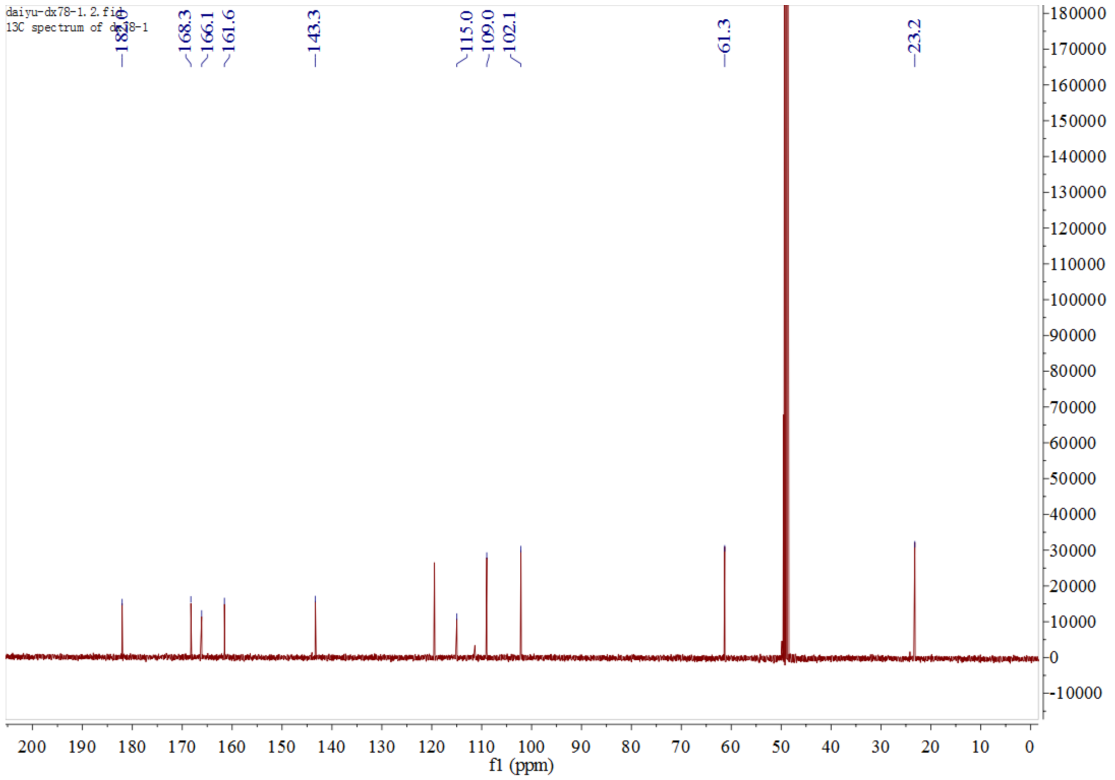

Figure S28. The ^{13}C-NMR spectrum of **4** in CD_3OD

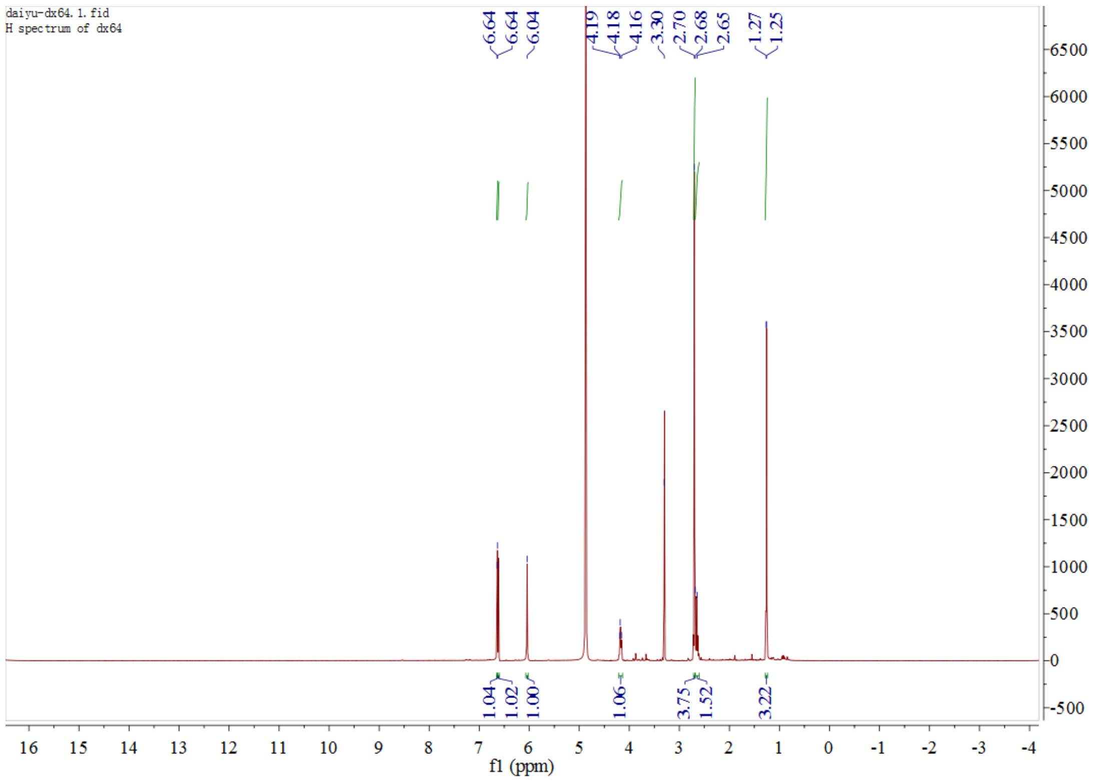

Figure S29. The ^{1}H-NMR spectrum of **5** in CD_3OD

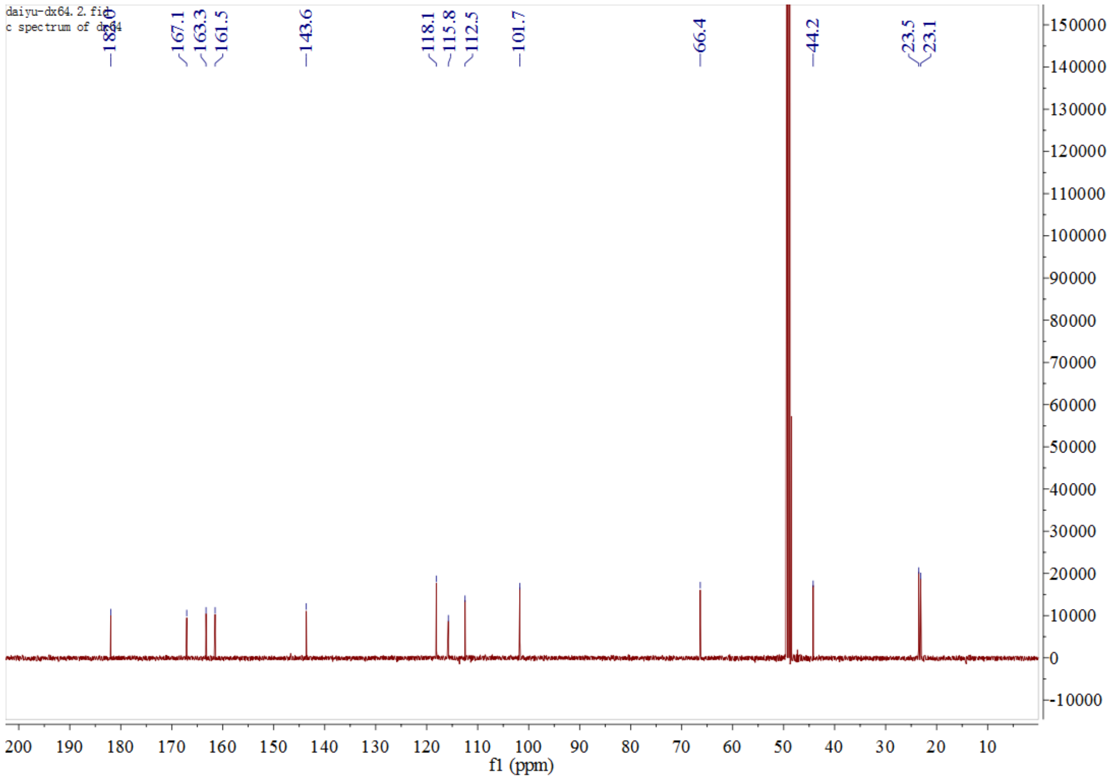

Figure S30. The ^{13}C-NMR spectrum of **5** in CD_3OD

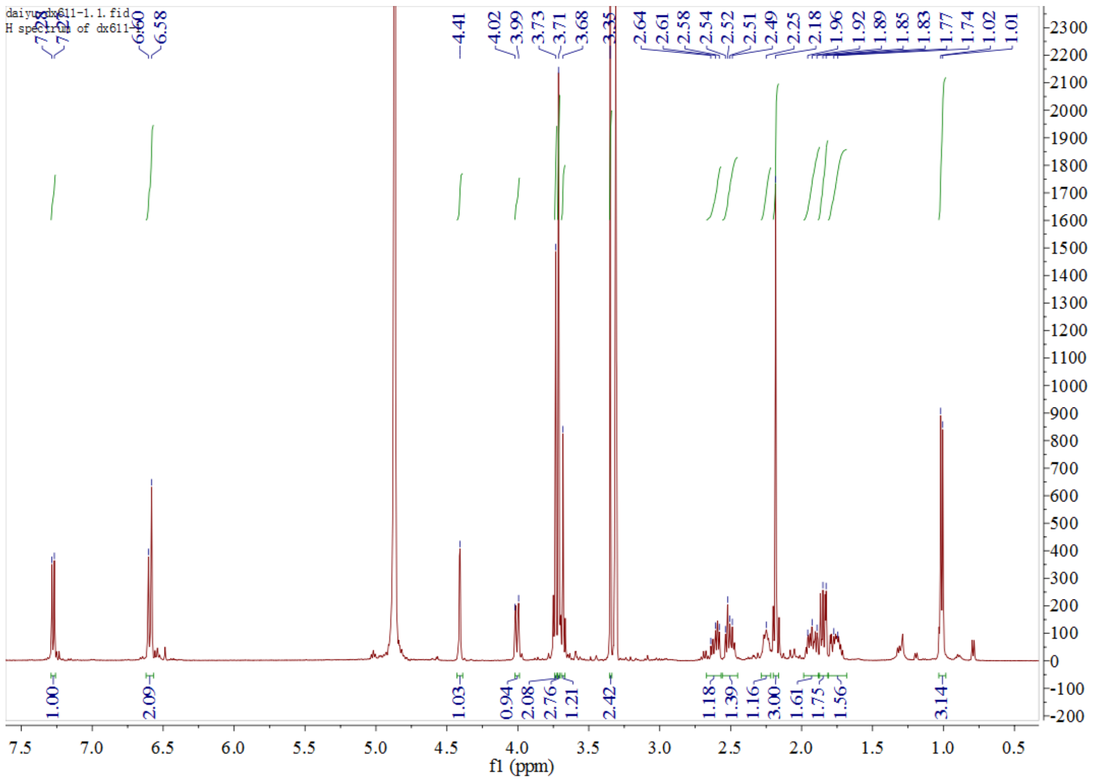

Figure S31. The ¹H-NMR spectrum of **6** in CD3OD

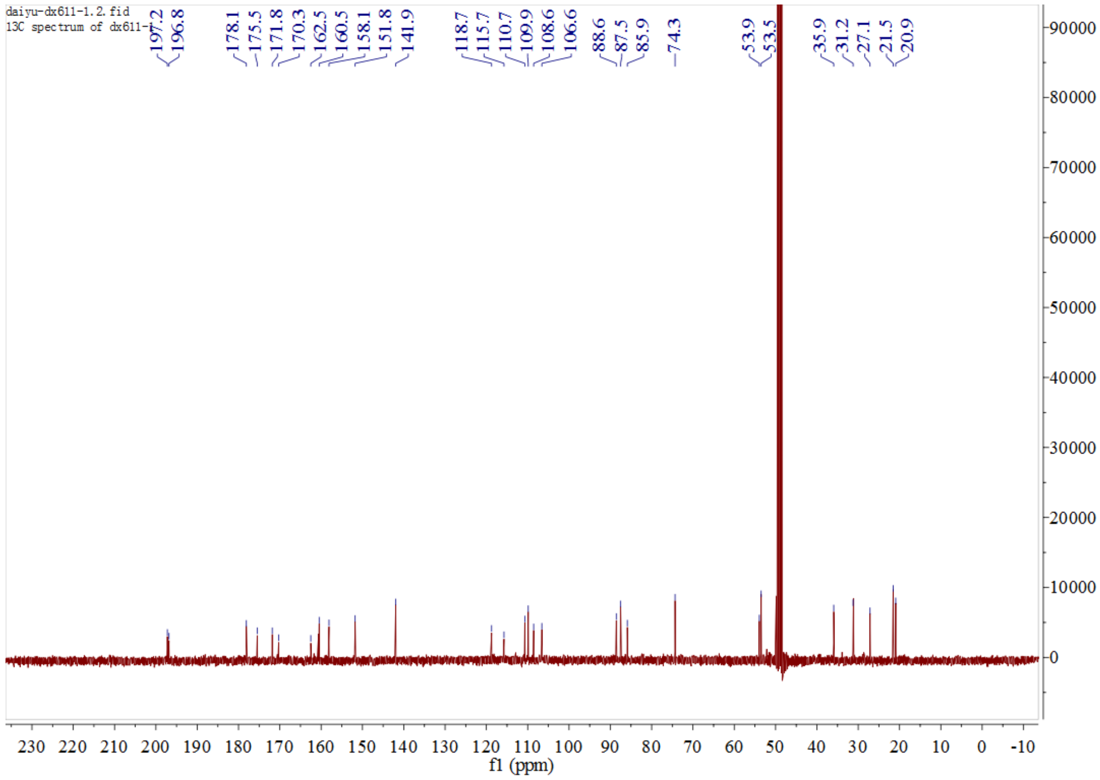

Figure S32. The ¹³C-NMR spectrum of **6** in CD3OD

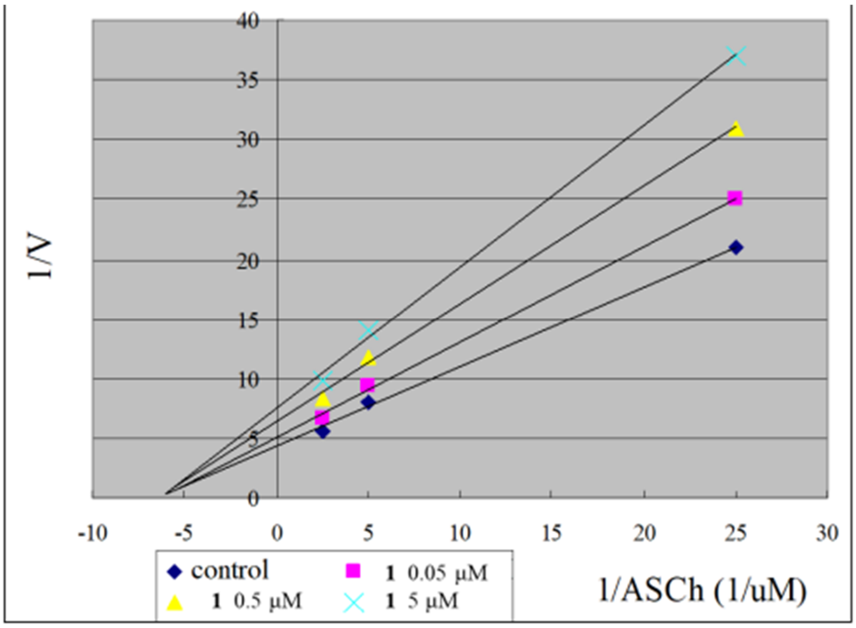

Figure S33. Preliminary Lineweaver–Burk plots for the AChE inhibition with **1**
